# Laparotomy and endoscopy cooperative resection in the treatment of Peutz-Jeghers syndrome

**DOI:** 10.1055/a-2665-7314

**Published:** 2025-08-20

**Authors:** Qing Li, Xiangbing Deng, Yao Yi, Feifan Chen, Xue Xiao

**Affiliations:** 1 34753 Department of Gastroenterology and Hepatology, West China Hospital, Sichuan University, Chengdu, Sichuan, China; 2 34753 Sichuan University-University of Oxford Huaxi Joint Centre for Gastrointestinal Cancer, Department of Gastroenterology and Hepatology, West China Hospital, Sichuan University, Chengdu, China; 3 34753 General Surgery Department 2, Colorectal Gastrointestinal Surgery, West China Tianfu Hospital, Sichuan University, Chengdu, Sichuan, China; 4 Colorectal Cancer Center, West China Hospital, Sichuan University, Sichuan, China

In Peutz–Jeghers syndrome (PJS), laparotomy is recommended for patients accompanied by intestinal intussusception or malignancy 1 2 3 . Resection of the remaining asymptomatic polyps during operation is unusual, with increased risk of enteric fistula or short bowel syndrome, while the following endoscopic polypectomy may be tough. We considered that laparotomy and endoscopy cooperative resection might be safe and efficient to solve the problem.

A 25-year-old man presented with chronic abdominal pain for 8 months. He underwent jejunectomy and total colectomy because of PJS-induced obstruction in childhood; there were 3.6 m of intestine retained ( Fig. 1 ). Single-balloon-assisted enteroscopy (SBE) showed hundreds of polyps (pedunculated or thick stalk) ranged from 0.5 to 4 cm in the intestine, with a large polypoid elevated lesion obstructing the ileal stump ( Fig. 2 ). Contrast-enhanced abdominal computed tomography suggested intussusception with concentric circle sign ( Fig. 3 ). LECR was then decided.

**Fig. 1:**
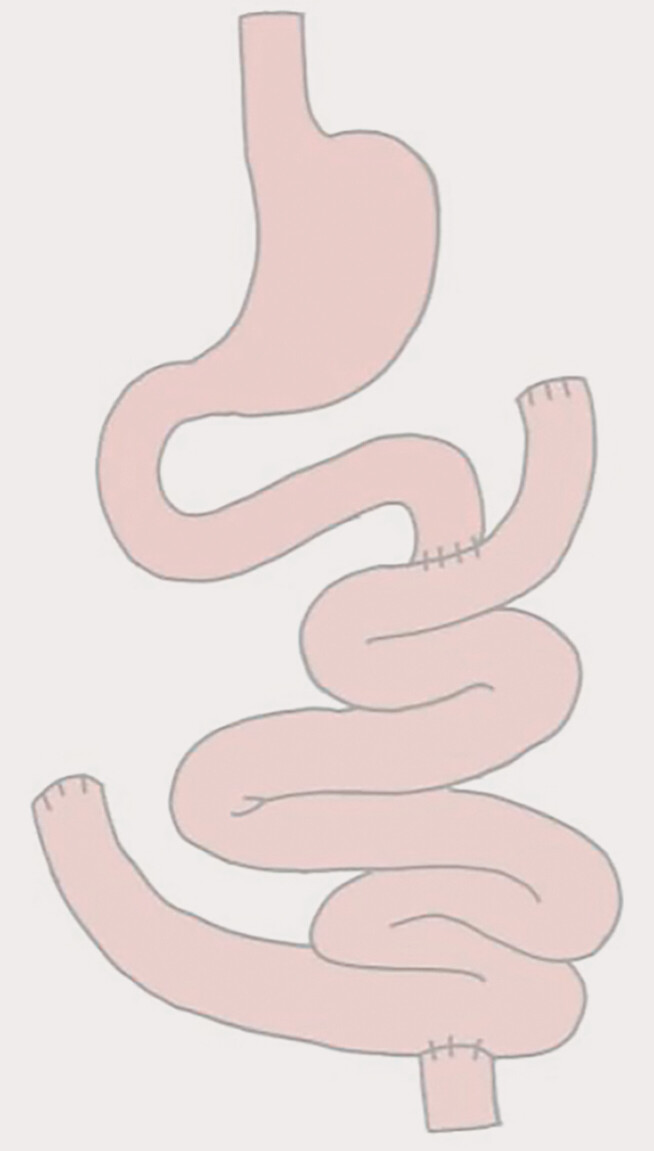
Changed anatomy of GI tract after subtotal colectomy and partial enterectomy.

**Fig. 2:**
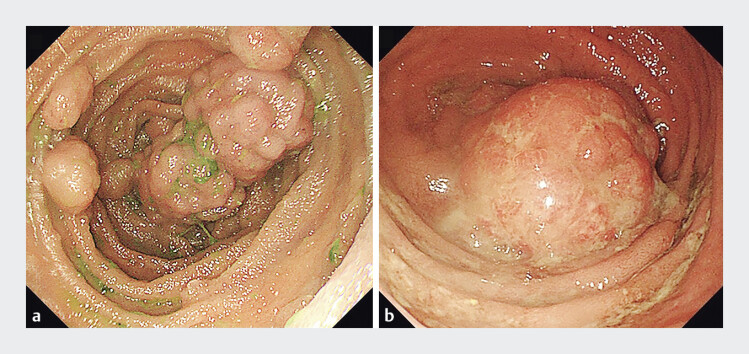
SBE showed hundreds of polyps ranged from 0.5 to 4 cm in the intestine ( **a** ), with a large polypoid elevated lesion obstructing the ileal stump ( **b** ).

**Fig. 3:**
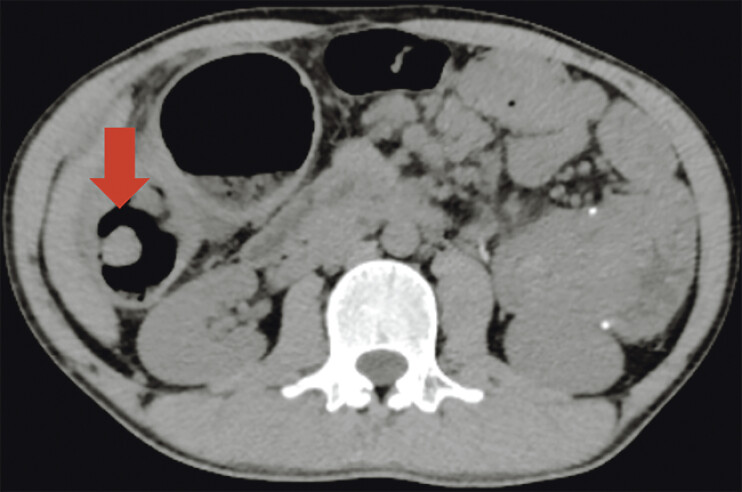
Abdominal CT showed “concentric circle sign” (arrowhead).

During laparotomy, the endoscopist utilized an Olympus PCF-260J endoscope, conducted surgeon-assisted transoral and transanal enteroscopic polypectomies ( Fig. 4 ). For polyps ≥1.5 cm, hot snare polypectomy was performed, followed by clip closure of the resection site. In cases demonstrating pulsatile bleeding at the wound base or exhibiting high-risk features of perforation, trans intestinal wall suturing was subsequently performed by the surgeon under endoscopic visualization ( Fig. 5 , Video 1 ). Also, the surgeon reduced the intussusception and removed the ileal stump. The patient experienced an uneventful postoperative recovery with no infection, delayed hemorrhage, or perforation and was discharged 1 week later.

**Fig. 4:**
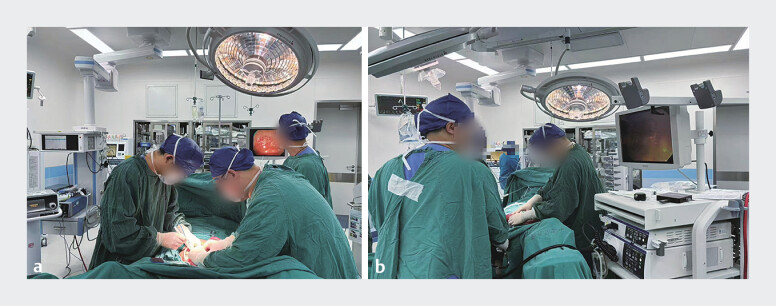
The endoscopist conducted surgeon-assisted transoral ( **a** ) and transanal ( **b** ) enteroscopic polypectomies.

**Fig. 5:**
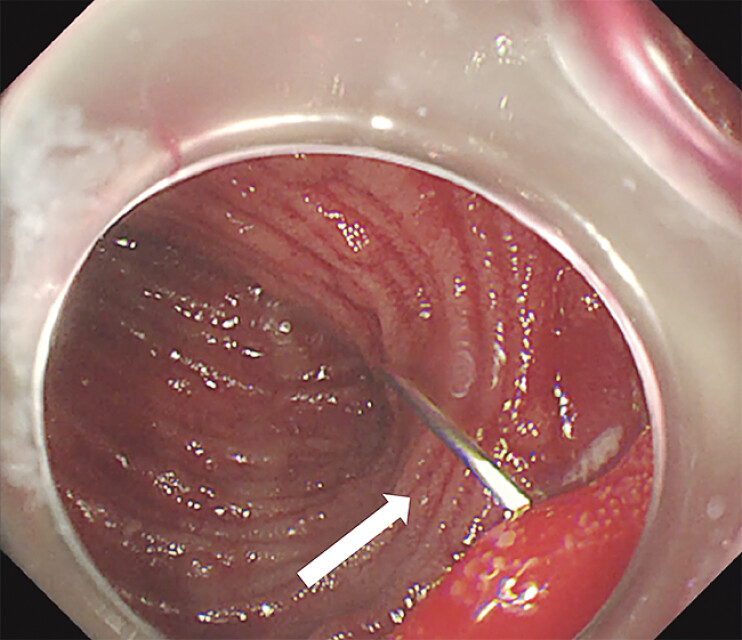
Trans intestinal wall suturing (arrowhead) was performed under endoscopic visualization.

Laparotomy and endoscopy cooperative resection in the treatment of Peutz–Jeghers syndrome.Video 1

Postoperative pathology revealed mucinous adenocarcinoma invading beyond the serosal layer of the ileal stump without lymph node metastasis. Adjuvant 5 cycles of chemotherapy combined with immunotherapy (XELOX regimen + bevacizumab) were applied. Ten months after LECR, the patient recovered very well without special symptoms. Follow-up SBE and CT revealed no evidence of cancer recurrence or metastasis. The remaining small intestinal polyps were successfully removed with SBE.

Endoscopy_UCTN_Code_TTT_1AT_2AD

## Notes

Endoscopy E-Videos https://eref.thieme.de/e-videos
*E-Videos* is an open access online section of the journal *Endoscopy* , reporting on interesting cases and new techniques in gastroenterological endoscopy. All papers include a high-quality video and are published with a Creative Commons CC-BY license. Endoscopy E-Videos qualify for HINARI discounts and waivers and eligibility is automatically checked during the submission process. We grant 100% waivers to articles whose corresponding authors are based in Group A countries and 50% waivers to those who are based in Group B countries as classified by Research4Life (see: https://www.research4life.org/access/eligibility/ ). This section has its own submission website at https://mc.manuscriptcentral.com/e-videos .
